# Effects of cardiac telerehabilitation during COVID-19 on cardiac hemodynamic and functional responses and quality of life: a randomized controlled trial

**DOI:** 10.1097/MS9.0000000000002235

**Published:** 2024-10-02

**Authors:** Mostafa Dehghani, Mostafa Cheraghi, Amir Shakarami, Morteza Dehghani, Mehrdad Namdari

**Affiliations:** a Department of Cardiovascular Research Center, Shahid Rahimi Hospital, Lorestan University of Medical Science, Khorramabad, Iran; b Department of Cardiovascular Research Center, Shahid Beheshti University of Medical Sciences, Tehran, Iran

**Keywords:** coronary artery disease, COVID-19, health-related quality of life, hemodynamic and functional responses, telerehabilitation

## Abstract

**Purpose:**

This study aimed to assess the effects of a home-based cardiac telerehabilitation (HBCT) on cardiac hemodynamic and functional responses and health-related quality (HRQOL) of the patients undergoing percutaneous coronary intervention (PCI).

**Materials and methods:**

In this randomized controlled clinical trial, single-blinded. One hundred-fifty-five patients (mean age: 50.41±7.3 years, 41 women and 39 men) who underwent PCI were randomized into the two groups of intervention and control. The HBCT program included supervised exercise training, walking, phone calls, and a pedometer for 8 weeks. Hemodynamic changes, including systolic blood pressure (SBP) and diastolic blood pressure (DBP), resting heart rate (HR_rest_), maximum heart rate (HR_max_), ejection fraction (EF), and rate pressure product (RPP), and functional parameters including the distance walked and metabolic equivalents (METS), also HRQOL were measured in all patients before and after the 8-week HBCT program.

**Results:**

Our results showed significant reductions in SBP_rest_ (126.82±9.17 vs. 131.27±10.24; *P* =0.044), DBP_rest_ (87.4±5.39 vs. 89.17±7.33; *P*=0.027), HR_rest_ (76.15±3.01 vs. 77.65±4.16; *P*=0.041), HR_max_ (143.1±5.24 vs. 147.57±8.63; *P*=0.011), and RPP (9.64±0.81 vs. 10.07±0.99; *P*=0.007) and significant elevations in (45.75±4.31 vs. 43.5±5.21; *P*=0.039), distance walked (514.95±214.5 vs. 368.04±221.43; *P*=0.019), Mets (7.41±0.84 vs. 6.89±1.28; *P*=0.018), as well as HRQOL in the MCS (50.62±10.45 vs. 46.25±7.74; *P*=0.037), and HRQOL in the PCS (46.75±8.73 vs. 42.37±9.99; *P*=0.040) in the intervention group compared to the control group.

**Conclusion:**

An HBCT program consisting of supervised exercise training significantly improved hemodynamic response, exercise performance capacity, and HRQOL in patients following PCI.

## Introduction

Highlights

- Amid the COVID-19 pandemic, many outpatient health meditation services for example, cardiac rehabilitation were postponed for safety reasons; so, an alternative CR transfer strategies should be used to overcome these barriers.
- Telerehabilitation, as an alternative approach to removeing some of these barriers, includes providing distance rehabilitation services via data and connection technologies such as mobile and Internet.
- The implementation an CRP-supervised telerehabilitation program consisting of supervised exercise training significantly improved hemodynamic response, exercise performance capacity in patients during the COVID-19 pandemic.
- Performing the CRP-supervised telerehabilitation more effectively improved health-related quality of life in patients.

Coronary artery disease, one of the most common cardiovascular diseases (CVDs), accounts for a large proportion of deaths worldwide^1^. The results of numerous studies show that performing exercise-based cardiac rehabilitation (CR) promotes the health, prognosis, and HRQOL of cardiovascular patients^2^.

On the other hand, the entire world was ravaged by the SARS-CoV-2 virus, the causative agent of the COVID-19 pandemic, in 2020^3^. Amid the COVID-19 pandemic, many elective and outpatient health meditation services were postponed for safety reasons. Currently, the in-person services of rehabilitation centers around the world are almost stagnant and half closed due to the COVID-19 infection^4^ these programs are partially or wholly interrupted in many centers and specialized CR clinics. Many countries have instructed people to stay at home^5^, which brings severe problems to patients with cardiovascular diseases, especially the elderly, who need regular physical activity. Recently, more flexible alternatives have been developed that facilitate access to and participation in CRPs. This is the case of HBCT. The most well-known of these and the only one validated by the National Institute for Health and Clinical Excellence in the UK is the Heart Manual program^6^. The authors suggest that hospital-supervised CRP was chosen based on access to specialists, the availability of more sophisticated individualized programs, and the patient’s perceived sense of safety when in a center. This sense of safety in supervised CRP is well-attested and is due to correct risk stratification. Thus, the patient can be offered an individualized training program, and the degree of supervision required during the CRP can be assessed^7^. An alternative CR transfer strategies should be used to overcome these barriers. Telerehabilitation, as an alternative approach to removing some of these barriers, includes providing distance rehabilitation services via data and connection technologies such as mobile, the internet, and pictorial^8^. This pattern has been successfully implemented for people with different cardiovascular patients and is now being promoted as a component of pattern healthcare^9^. Therefore, one of the ways to ensure continuous care for cardiovascular patients is to integrate CR programs with remote medical devices (such as pedometers) in the context of controlled, monitored home-based plans to reduce the risk of COVID-19 transmission and overcoming barriers related to treatment costs^10^, and increase improve physical capacity.

In this randomized clinical trial, the effects of home-based CR compared to routine care CR on cardiac hemodynamics, functional responses, and HRQOL in coronary artery disease patients during the COVID-19 pandemic. We also hypothesized that telerehabilitation would be as effective as traditional CR realized in a conventional hospital setting.

## Methods

### Material and methods

**Figure 1:**
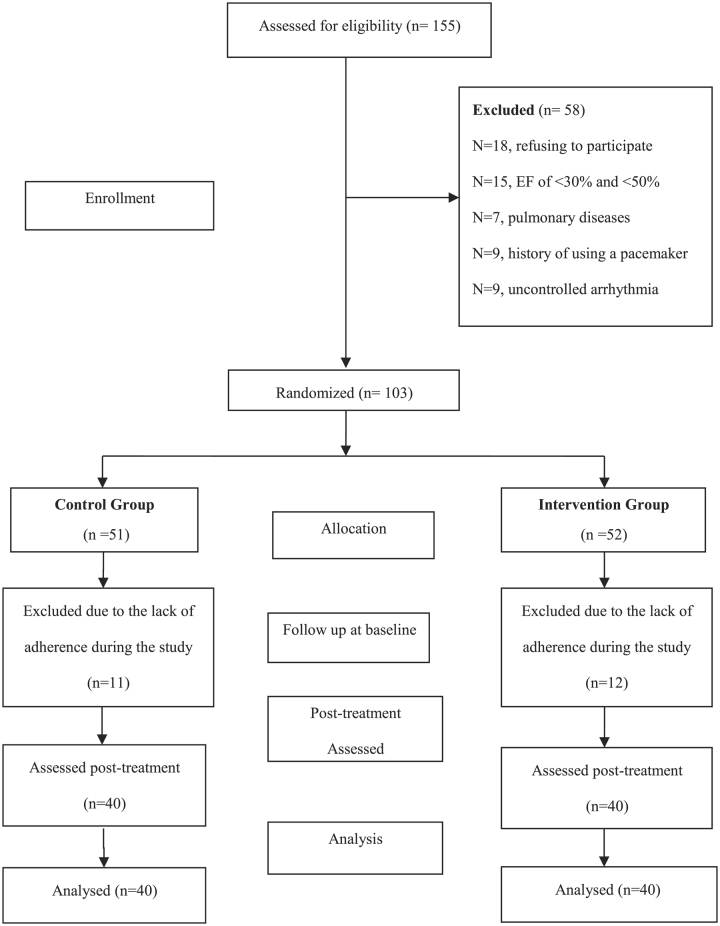
CONSORT flowchart of the trial.

This was a randomized single-blinded controlled trial conducted in line with CONSORT criteria (Fig. 1). Our aim was to examine the effects of an 8-week CR (performed in home-based CR compared to routine care CR) on cardiac hemodynamic, functional responses and HRQOL in patients with CVDs, no previous history of pulmonary infection following percutaneous coronary intervention (PCI). The patients were initially examined by a physician to determine their heights and weights (Xiaomi Mi Body Composition 2 769 Scale) using standard methods. The BMI and by dividing weight (kilograms) by the square of the body height measured in meters (kg/m^2^). Each participant then sat calmly for 10 min, and ECG 12 (Kenz Cardio 601, Suzuki Co.) was performed to determine the rest heart rate (HR_rest_). Then systolic and diastolic blood pressures at rest (Beurer BM-16 Blood Pressure Monitor) were recorded.

### Experimental design

This was a randomized, single-blinded controlled trial to examine the effects of an 8-week CR program on the hemodynamic responses and functional capacities of patients with CAD following PCI. All patients with acute CAD, at least 2 months passing from PCI, admitted to the ICU of the cardiology ward of the Cardiovascular Research Center of the Shahid Rahimi Hospital, and discharged after PCI between October and November 2020, were enrolled in this study. All the methods employed in this research were registered at the Iranian Registry for Clinical Trials under the code IRCT20181122041725N2. Eligible patients were assigned to two groups: home-based CR (telerehabilitation) and traditional center-based CR (conventional CR). Each group was composed of 40 patients (Fig. 1). In this study, the assessment procedures were implemented in a two-step framework (before and after the 8-week CRP). Overall, 40 CR sessions were scheduled over 8 weeks (three sessions per week)^11^ by allocating a predefined time to each exercise period. All the patients of the intervention group also received psychological, nutritional, and smoking cessation consultations. In addition, weekly educational sessions were held for all patients during the study, including explanations about cardiovascular diseases, their risk factors, diagnostic and therapeutic approaches, and their medications and complications, as well as stress reduction methods, and the importance of a healthy lifestyle (quitting smoking, taking healthy diets, and doing regular physical activities). The patients who agreed to participate provided a telephone number and designated a time appropriate for follow-up phone calls. Formal consent was also obtained over the telephone during data collection.

### Inclusion criteria

Adults with complete revascularization after PCI (with the culprit vessel-only approach) and LV systolic dysfunction were included in the study. In addition, inclusion criteria were the use of aspiration thrombectomy or intravenous glycoprotein IIb/IIIa antagonists, also were an ejection fraction (EF) between 30 and 50%, the diagnosis of mild to moderate cardiovascular disorder (New York Heart Association classes II and III)^12^, age of 45–60 years, and living in the city (because living in urban areas is more sedentary than in rural areas due to less time for physical activities due to traffic, etc., resulting in a higher mortality rate)^13^. Also, those with severe ventricular dysfunction (ejection fraction <30%), refusal to give informed consent, recurrent ischemia, no history of using a pacemaker, concurrent pulmonary diseases or severe chronic respiratory disease, and uncontrolled arrhythmia were excluded from the study. The study was approved by the Ethics Committee of Lorestan University of Medical Sciences (approval ID: IR.LUMS.REC.1399.199).

Exclusion criteria after explaining the content and timing of the cardiac rehabilitation program and its necessity, the patients who were willing to participate in the study were requested to sign an informed consent form. One hundred and fifty-five patients were initially recruited, of whom 102 eligible individuals were allocated into the intervention and control groups via the permuted block randomization method. The patients who attended less than 25% of rehabilitation sessions (lack of adherence) were excluded from the study (*N*=58, Fig. 1).

### Cardiac telerehabilitation

Before the start of the CR plan, the walking program was handed over to the patients of the intervention group in a printed notebook to record the number of steps based on the pedometer feedback and supervised exercise training usually lasted 4–6 min and was made by the CR physician twice weekly. The patients were also requested to contact the researcher if they had any symptoms (e.g. chest pain, significant arrhythmias, etc.) during exercises, or if they had intention to withdraw from the study. The subjects in the control group were received a recommendation from the physician to walk 30–40 min (five times a week), the physical exercise program, the frequency, and the intensity were the same for the control CR group, except they did not have any supervised exercise training during this period. The participants signed a written informed consent form before entering the study. During the study, all the participants (the intervention and control groups) received their usual medications. In this study, first, we determined the pattern of carrying out progressive exercises and ascertained the number of steps to be taken at the start. From the second to the eighth week, the number of steps gradually increased by 15% per week (during 30 min of walking activity (Table 1), along with an increase in load and intensity up to 70–85% of the heart rate)^14^. Each home-based session lasted about 50 min; the first 10 min included a warm-up, followed by 30 min of aerobic walking on a flat surface using a pedometer (Rossmax PA-W55), and at the end, a 10 min cooling down. The perceived exertion was rated on the Borg dyspnea scale. This program was designed based on a previous study^15^.

**Table 1:** Rehabilitation program is based on the number of steps (using a pedometer) in the first to eighth weeks (intervention group)

Weeks	First	Second	Third	Fourth	Fifth	Sixth	Seventh	Eighth
Number of steps per day	3000^a^	3750	4500	5250	6000	6750	7500	8250

a During consecutive weeks, 150 steps per day were added to the number of steps in the same period of 30 min as an overload.

### Assessments

As mentioned, the assessment procedures were implemented in a two-phase framework (i.e. before and after the 8-week CR program). Also, before starting the CR program, left ventricular ejection fraction (LVEF) was measured by two-dimensional (M-mode) echocardiography, and the patients were stratified (low, intermediate, and high-risk) based on the results of the exercise test and LVEF evaluation. The rate pressure product (RPP), which is used by cardiovascular physiologists to determine the cardiac stress level, internal load, and myocardial oxygen consumption, was calculated by multiplying the resting systolic blood pressure (SBP) by rest HR, divided by 100 (i.e. RPP=SBP × HR/100). The RPP is a parameter used to estimate the risk of ischemic events leading to infarction^15^.

The participants were asked to avoid taking alcohol and caffeine and smoking 24 h before the exercise test. The advanced treadmill test was conducted using the modified Bruce protocol to determine functional responses (Mets and the distance walked)^11^. The predicted peak heart rate was calculated by deducting the patient’s age from 220. The participants were encouraged to exercise until feeling limited symptoms, even if 85% of the maximum predicted heart rate was achieved^16^. The criteria for exercise termination included physical exhaustion or reaching a maximum heart rate more significant than the value obtained by the ‘age-predicted maximum’ formula. During each exercise and recovery phase, symptoms, blood pressure, and heart rate were recorded. After reaching the peak exercise threshold, participants walked for 2 min (1.5 mph, 2.5% grade) to cool down. At the end of the intervention period, the exercise test and echocardiography were performed again before and after the CRP was for all patients.

### Assessment of health-related quality-of-life

HRQOL is an outcome of healthcare and a consequence of illness, that is scarce or ambiguous^17^. Therefore, instrumentals to distinguish HRQOL have become necessary result measures for the evaluation of healthcare providers. The last few decades of HRQOL instrumentals, are increasingly being incorporated in clinical trials. The HRQOL was assessed using the Medical Outcomes Study 36-item Short Form Health Survey (SF-36). The SF-36 contains 36 sections grouped into eight primary domains that constitute two ingredients. Therefore, physical functioning (PF), role-physical (RP), bodily pain (BP), and general health (GH) constitute the physical health component, vitality (VT), social functioning (SF), role-emotional (RE), and mental health (MH) constitute the psychic health part. The scores are then transformed into a zero to a hundred numeric measure. The eight major were scored on a step from zero to a hundred points, indicating the worst to best possible health, respectively^18^. This questionnaire evaluates different physical and psychological aspects of quality of life. The highest score in each of the eight indices is 100 (the best possible situation) and the lowest score is zero (the worst possible situation)^19^. Scores for all domains were further summarized and standardized into the physical health component score (PCS). The mental health component score (MCS), according to a user manual, with higher scores showing a better HRQOLs^18^. The number of HRQOL was summarized as the MCS and the PCS. MCS index, which was the sum of the number of mental health component scores a PCS index, which was the sum of the number of physical health component scores.

### Statistical analysis

SPSS 22 (IBM Inc.) was used for statistical analyses. All continuous variables were expressed as mean±SD, and categorical variables were expressed as number (*n*) and percentage (%). The samples *t*-test, paired *t*-test, and *χ* ^2^ test were used to determine significant differences in variables between or within the intervention and control groups. The statistical significance level (alpha) was set at *P*<0.05. The sample size was determined according to a previous study^20^ and using the formula designed for comparing the means of two independent populations. Considering a CI of 95% (alpha error rate of 5%), the beta error of 20%, and power of 80%, an allocation ratio of 1:1; and analysis requiring two pairwise comparisons, the sample size of each group was calculated *n*=40 ( $Z\left(1-\beta \right)=0.84$ , $Z\left(1-\frac{\alpha }{2}\right)=1.96$ , s1=s2=1.4, x2=8.5, and x1=9.25).


$$n=\frac{2(Z\left(1-\frac{\alpha }{2}\right)+Z{\;{\left(1-\beta )\right)}^{2\;}\times S}^{2}}{{\left(x1-x2\right)}^{2}}.$$

### Randomization and blinding

First, we talked to the patients who had indications for undergoing rehabilitation about the advantages and disadvantages of home-based and in-hospital rehabilitation methods. Then the patients who were eligible for either method were randomized to the intervention and control groups using an allocation software through the block randomization method. In this method, the blocks were arranged randomly with letters A and B. We used blocks with sizes of 3, 6, and 4, so that the size of the tiles is the same. By combining the random blocks together, we created a balanced random list for the two treatment groups. Until the intended sample size in each group was reached, random allocation continued using the above blocks. This study was a single-blinded trial, where the physician (researcher) and the patient were aware of the type of the rehabilitation program; however, the statistician who collected and analyzed the data did not know whether the patient was rehabilitated at a supervised or unsupervised method.

## Results

All patients (103 participants) were randomly registered in this clinical trial. Overall, 80 patients with PCI were randomly divided into the control (*n*=51) and intervention (*n*=52) groups. The intervention group consisted of 51 patients (25 men and 26 women) with a mean age of 49.77±7.88 years, and the control group included 52 patients (27 men and 25 women) with a mean age of 51.45±7.46 years. The patient’s demographic and clinical characteristics are shown in Table 2. There were no significant differences comparing the demographic data, hemodynamic responses, functional capacity, and HRQOL between the two groups (Table 2). Hemodynamic and functional parameters in the study groups have been presented in Table 3.

**Table 2:** Baseline characteristics of the subjects in this research

Variables	Intervention group (*N*=40)	Control group (*N*=40)	*P*
Age (years)	49.77±7.88	51.45±7.46	0.33
Sex (men/women)	19/21	20/20	—
BMI(Kg/m^2^)	24.91±1.68	25.63±1.86	0.07
Smoking	1(2.5%)	1(2.5%)	> 0.99
Hypertension	4(10%)	2(5%)	0.16
Family history of heart disease	3(7.5%)	5(12.5%)	0.46
History of hyperglycemia	2(5%)	3(7.5%)	0.71
PTCA	4(10%)	5(12.5%)	0.66
Myocardial infarction	4(10%)	5(12.5%)	0.66
Diabetes mellitus	2(5%)	1(2.5%)	> 0.99
NSTEMI	4(10%)	3(7.5%)	0.56
STEMI	5(12.5%)	5(12.5%)	> 0.99

Values expressed as mean±SD, number or percentage.

NSTEMI, non-ST-segment elevation myocardial infarction; PCI, percutaneous coronary angioplasty; PTCA, percutaneous transluminal coronary angioplasty; STEMI, ST-segment elevation myocardial infarction.

*Significant difference at *P*≤0.05.

**Table 3:** Within-group and between-group variations in hemodynamic parameters, functional responses, and health-related quality of life before and after the cardiac rehabilitation program in the two studied groups

	Intervention group (*N*=40) (Mean±SD)			Control group (*N*=40) (Mean±SD)			
Hemodynamic, functional responses, and HRQoL	Pre	Post	P^a^	Pre	Post	P^b^	P^c^
SBP (rest) (mmHg)	134.4±8.69	126.82±9.17	<0.001***	130.17±11.78	131.27±10.24	0.257	0.044*
DBP (rest) (mmHg)	92.9±6.98	87.4±5.39	<0.014*	89.82±7.57	89.17±7.33	0.200	0.027*
HR (rest) (beat/min)	78.27±3.21	76.15±3.01	0.021**	78.02±4.41	77.65±4.16	0.34	0.041*
HR (max) (beat/min)	148.32±6.28	143.1±5.24	<0.011**	146.75±7.59	147.57±8.63	0.654	<0.001***
EF (%)	43.5±5.21	45.75±4.31	0.002**	43±6.18	43.5±5.21	0.160	0.039*
RPP	10.46±0.83	9.64±0.81	0.021*	10.1±0.83	10.07±0.99	0.689	0.007**
Distance walked (m)	369.02±146.74	514.95±214.5	<0.001***	417.15±209.06	368.04±221.43	0.254	0.019**
Mets (%)	7.09±0.89	7.41±0.84	0.022*	6.61±1.39	6.89±1.28	0.060	0.018*
HRQOL							
MCS	46.75±10.47	50.62±10.45	0.029*	44.50±9.8	5 46.25±7.74	0.217	0.037*
PCS	43.25±7.72	46.75±8.73	0.011*	41.50±8.63	42.37±9.99	0.544	0.040*

*Significant difference: *P*<0.05.

DBP, diastolic blood pressure; EF, ejection fraction; HR, heart rate; HRQOL, health-related quality of life; MCS, mental health component score; PCS, physical health component score; RPP, rate pressure product; SBP, systolic blood pressure.

^a^ P and.

^b^ P value.

^c^ P value.

*Significant difference at the intervention and control groups compared with before and after cardiac rehabilitation program, using paired samples *t*-test.

*Significant difference at the intervention and control groups after cardiac rehabilitation program, using ANCOVA.

* *P*<0.05.

** *P*<0.01.

*** *P*<0.001.

**Figure 2:**
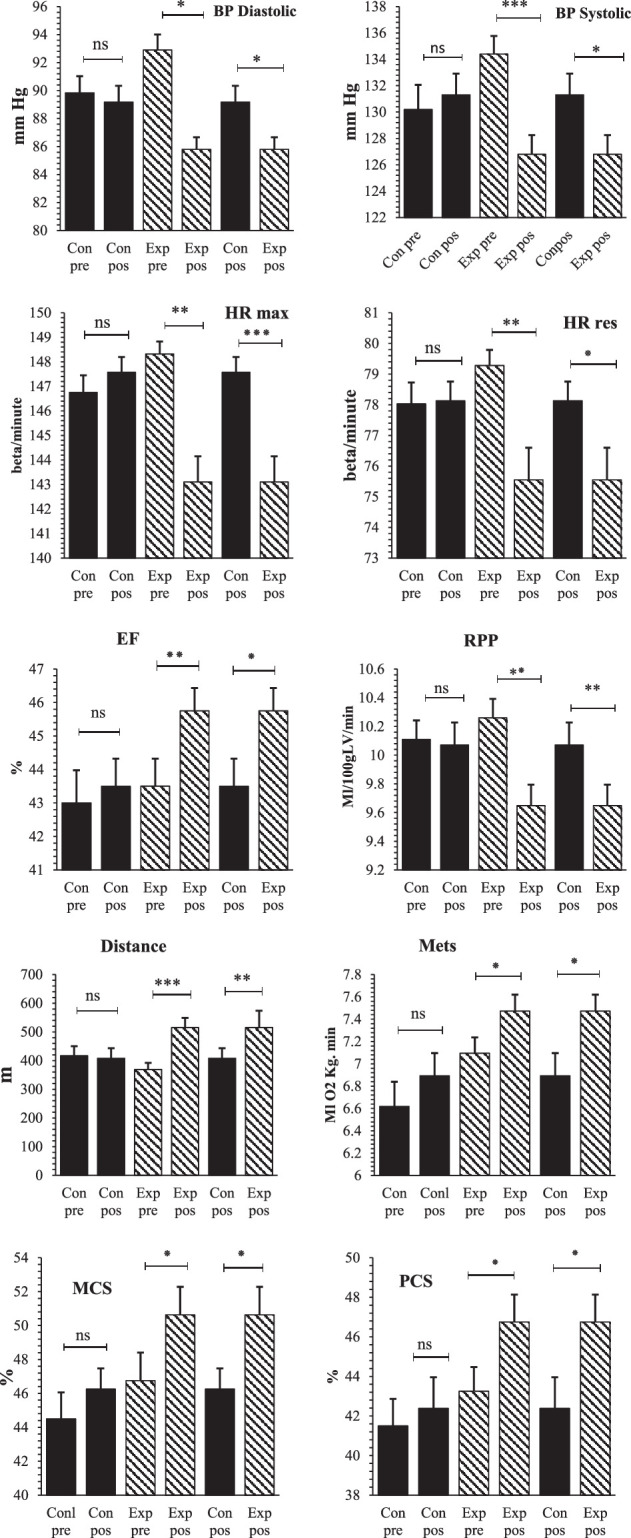
Within-group and between-group variations in hemodynamic parameters, functional responses, and health-related quality of life before and after the cardiac rehabilitation program in the two studied groups. ^*^Significant difference: *P*<0.05; DBP, diastolic blood pressure; EF, ejection fraction; HR, heart rate; MCS, mental health component score; PCS, physical health component score; RPP, rate pressure product; SBP, systolic blood pressure. ^*^ *P*<0.05, ^**^ *P*<0.01, ^***^ *P*<0.001.

Regarding within-group comparisons, significant differences were observed comparing hemodynamic parameters and functional responses, including significant decreases in SBP_rest_ (from 134.4±8.69 to 126.82±9.17 mmHg, *P*=0.001), DBP_rest_ (from 92.9±6.98 to 87.4±5.39 mmHg, *P*<0.014), HR_rest_ (from 78.27±3.21 to 76.15±3.01 beat/min, *P*=0.021), HR_max_ (from 148.32±6.28 to 143.1±5.24 beat/min, *P*=0.011), and RPP (from 10.46±0.83 to 9.64±0.81, *P*=0.021) and significant increases in EF (from 43.5±5.21 to 45.75±4.31%, *P*=0.002), distance walked (from 369.02±146.74 to 514.95±214.5 m, *P*=0.001), Mets (from 7.09±0.89 to 7.41±0.84%, *P*=0.001), also HRQOL in the MCS (from 46.75±10.47 to 50.62±10.45%, *P*=0.029), and HRQOL in the PCS (from 43.25±7.72 to 46.75±8.73%, *P*=0.011) in the experimental group before and after the 8-week HBCT program (Table 3 and Fig. 2).

After the 8-week HBCT program, significant differences were observed comparing hemodynamic parameters, functional responses, and HRQOL between the two groups, including significantly lower SBP_rest_ (126.82±9.17 vs. 131.27±10.24; *P*=0.044), DBP_rest_ (87.4±5.39 vs. 89.17±7.33; *P*=0.027), HR_rest_ (76.15±3.01 vs. 77.65±4.16; *P*=0.041), HR_max_ (143.1±5.24 vs. 147.57±8.63; *P*=0.011), and RPP (9.64±0.81 vs. 10.07±0.99; *P*=0.007) and significantly higher EF (45.75 ± 4.31 vs. 43.5±5.21; *P*=0.039), distance walked (514.95±214.5 vs. 368.04±221.43; *P*=0.019), Mets (7.41±0.84 vs. 6.89 ± 1.28; *P*=0.018), as well as HRQOL in the MCS (50.62±10.45 vs. 46.25±7.74; *P*=0.037), and HRQOL in the PCS (46.75 ± 8.73 vs. 42.37±9.99; *P*=0.040) in the experimental group (Table 3 and Fig. 2). However, no significant differences were observed in the mentioned hemodynamic parameters, functional responses, and HRQOL in the control group before and after the 8-week CR program.

## Discussion

This randomized controlled trial was performed to assess the effects of an HBCT program on hemodynamic parameters, cardiac functional capacity, and HRQOL in CAD patients after PCI. The exercise test revealed an increase in cardiac functional capacity after the implementation of the HBCT program. We also found that the 8-week supervised CR program (track telephone follow-up calls and use a pedometer) significantly improved the patients’ hemodynamic, functional, and HRQOL indices compared to the control.

Maintaining public health is the primary interest of all countries during the COVID-19 pandemic. Nevertheless, it is essential to develop clear recommendations on how to proceed with rehabilitation programs for people diagnosed with CAD. The practical implementation of CR strategies based on the gold standard is currently problematic due to the pandemic, which has led to the limitation or cessation of CR programs across the globe^4^. In many countries, traditional centers-based cardiac rehabilitation programs have been suspended due to the concrete measures adopted to flatten the COVID-19 pandemic curve. So, there is a crucial need for alternative CR approaches. The COVID-19 pandemic has urged medical centers to use innovative strategies to provide healthcare^21^. In this regard, HBCT can be used as an effective alternative to fill this gap^22^.

Patient safety is an essential issue when performing telerehabilitation and must be addressed. The studies have confirmed the safety of telerehabilitation exercise programs and HBCT in patients with CAD^23^, even in high-risk individuals, considering all the indications and contraindications of such exercises. During HBCT, the patient’s adherence to instructions can be strictly supervised via interactive cooperation between the participant and the telemonitoring center^23^. In our study, we did not record any adverse events during physical exercises, indicating the safety and feasibility of the telerehabilitation method.

A meta-analysis showed that SBP, DBP, and mean blood pressure significantly decreased compared to pre-exercise^24^. Also, home-based CR after MI was effective in preventing left ventricular systolic and diastolic dysfunction and improving cardiac function^25^. A study showed that a short-term home-based program significantly improved left ventricular function and EF in patients with acute myocardial infarction (MI) within 4 weeks^25^. However, the effect of CR on ventricular regeneration, especially in patients with low ejection fractions, is still controversial^26^. In this study, we provided an HBCT program to CAD patients undergoing PCI. We showed that the patient’s functional capacity significantly improved, indicating the beneficial effects of HBCT and physical activity on left ventricular function. As mentioned, HBCT significantly improved hemodynamic responses [SBP_rest_, DBP_rest_, HR_rest_, EF, RPP, the distance traveled (walked) on a treadmill, and metabolic equivalent of task (Mets); *P*<0.05] in the intervention group compared to the control group. Consistently, 8 weeks of a CR program was reported to have positive effects on the SBP_rest_, DBP_rest_, exercise tolerance, HR, and quality of life of patients with cardiac disease^27^.

The favorable regulatory effects of CR exercises on blood pressure can be due to improved autonomic (i.e. increased parasympathetic compared to sympathetic activity) and endothelial functions, as well as vasodilatory impacts^28^. In addition, another study reported the beneficial effects of exercises in patients with angina pectoris, evidenced by significant decreases in HR and SBP, leading to a decrease in RPP and an increase in workload under maximum activity^29^. The increased workload and the reduction in RPP, could indicate adequate myocardial oxygen consumption and good left ventricular function in patients, which was consistent with our results.

In the present study, we evaluated the effectiveness of a home-based CR program in PCI patients. Although numerous studies confirm our results, the effects of CR programs on central hemodynamic functions are not well-known. In another study, no significant impact was observed for a combined exercise program on the hemodynamic responses of males patients with CAD. This discrepancy may be because all participants in the recent study were males, and they had not been randomly allocated to the experimental groups, as well as due to different rehabilitation programs^30^.

Also, our results showed that the exercise tolerance threshold (as shown in the Bruce method) increased following the CR program, indicating an improvement in patients’ aerobic capacity. Consistent with our findings, the results of other studies showed that 8 weeks of CR at home significantly improved cardiovascular functional ability, metabolic activity, maximum oxygen consumption, and the exercise tolerance threshold on a treadmill in MI patients^16^. These changes often correlate with adaptations such as increased blood volume and EF, decreased vascular resistance, and increased skeletal muscles’ oxidative capacity^23,31^.

In conclusion, regular physical activity can maximize workload tolerance by reducing cardiac muscle contractions, cardiac output, and myocardial oxygen consumption. These events ultimately improve myocardial function and increase the body’s oxygen absorption and cardiac output and stroke volume, improving oxygen transfer and reducing cardiac stress and workload^31^. In addition, exercise can dilate coronary arteries and promote cardiovascular adaptations by regulating hemodynamic parameters such as SBP, DBP, HR, EF, and RPP and improving cardiac perfusion by correcting endothelial dysfunction^32^. Finally, our findings highlight the importance of HBCT along with supervised exercises and telephone follow-up calls in enhancing the lifestyle of the patients undergoing PCI amid the COVID-19 pandemic.

As, the results of this study showed that the implementation of the HBCT program amid the COVID-19 pandemic significantly improved different dimensions of HRQOL in PCI patients (*P*<0.05), which agreed with the observations of previous studies reporting that home-based cardiac rehabilitation compared to conventional methods could improvement in MCS and PCS components quality of life in patients undergoing percutaneous coronary intervention.

### Study limitations

The limitation of the present study was the lack of the assessment of systemic inflammatory markers, which generally elevate after PCI. Also, LV diastolic filling ways, assessed by transmitral Echo-Doppler, could have been influenced by a variety of factors such as heart rate and cardiac conduction, system loading conditions, valvular insufficiency, transmitral pressure gradient, and viscoelastic properties of the myocardium. In addition, this study was conducted in the city region. So, the distribution of the results of this study to other patients peoples should be attentively considered.

## Conclusions

HBCT effectively improved cardiac hemodynamic parameters, functional responses, and HRQOL in patients with low-moderate risk CAD. Considering the advancements in telemedicine, HBCT, as a remote patient-oriented program, can be regarded as a safe, effective, and standard home-based alternative to center-based CR during the COVID-19 pandemic. Wearable sensors can reliably transmit data to remote monitoring centers via data and connection technologies, providing CR specialists with the possibility of supervising the process, even over a global scope.

## Impacts on clinical practice

### What is known

The onset of the COVID-19 pandemic saw the suspension of center-based cardiac rehabilitation. Our results showed significantly effects of a home-based cardiac telerehabilitation program on cardiac hemodynamics, functional responses, and health-related quality of life of the patients undergoing percutaneous coronary intervention during the COVID-19 pandemic. Advantages of telerehabilitation approaches are their cost-effectiveness. Furthermore, no adverse events were reported during the program.

### What is new

Our research highlighted the promising role of data and connection technologies innovations in telemedicine, like and telephone follow-up calls during the COVID-19 pandemic.

## Ethical approval

Ethical approval for this study (Ethical Committee N° IR.LUMS.REC.1399.199) was provided by the Ethical Committee of Lorestan University of Medical Sciences, Lorestan, Iran on 15 November 2020.

## Consent

Written informed consent was obtained from the patients for publication and any accompanying images. A copy of the written consent is available for review by the Editor-in-Chief of this journal on request.

## Source of funding

Not applicable.

## Author contribution

M.D.: contributed to the study conception and design; M.C. and A.S.: material preparation, data collection, and analysis; M.D., M.C., M.D., and M.N.: helped training sessions and measurements; M.D. and M.N.: helped writing the paper. The first draft of the manuscript was written by MD and all authors contributed to the revisions of the manuscript. All authors read and approved the final manuscript.

## Conflicts of interest disclosure

The authors declare that they have no financial conflict of interest with regard to the content of this report.

## Research registration unique identifying number (UIN)

Registry URL: http://www.irct.ir. Iranian registry of clinical trial number: IRCT20181122041725N2.

## Guarantor

Mostafa Dehghani read and approved the final manuscript and accepts full responsibility for the work and/or the conduct of the study, had access to the data, and controlled the decision to publish.

## Data availability statement

All authors confirm any datasets generated during and/or analyzed during the current study are available, but, data sharing is not applicable to this article.

## Provenance and peer review

Not commissioned, externally peer-reviewed.
